# IS MIDDLE-AGED INEQUITY LIKELY TO PERSIST INTO OLD AGE?: TRAJECTORIES OF SOCIAL EXCLUSION IN KOREA

**DOI:** 10.1093/geroni/igad104.2327

**Published:** 2023-12-21

**Authors:** Jae Yeon Jung, Seok In Nam

**Affiliations:** Yonsei University, Seoul, Republic of Korea; Yonsei University, Seoul, Republic of Korea

## Abstract

Korea has the highest rate of older adult poverty among the OECD countries. The poverty of the older adult continues even with the government’s policies, as the loss of social roles, the reduction of the network, and the difficulty of escaping exclusion are complex. The goal is to find out how the trajectory of social exclusion changes as people get older. We used the data of the Korean Longitudinal Study of Ageing conducted by the Korea Employment Information Service. The target population is middle-aged people aged 45 to 52 (based on the first year), mainly the baby boom generation in Korea, and the total number of cases included in the analysis is 1,348. The trajectory of social exclusion change for 14 years from 2006 to 2020 was confirmed by composing on into economics, health, employment, housing, and social relationships. The group-based multi-trajectory model analysis was performed using Stata 16.1. The groups were identified as four: the low exclusion continuous maintenance group, the employment high exclusion maintenance group, the economic/housing high exclusion maintenance group, and the multi-domain high exclusion continuous maintenance group. All four types showed a tendency to maintain social exclusion from middle age until old age. Factors that caused differences between groups were gender, residential area, marital status, education, and income. These findings suggest that as people get older, the cumulative inequity in their lives becomes more pronounced. As a result, it will provide important clues for establishing policies for the welfare of older adults and alleviating social inequality.

